# An IFN-γ-IL-18 Signaling Loop Accelerates Memory CD8^+^ T Cell Proliferation

**DOI:** 10.1371/journal.pone.0002404

**Published:** 2008-06-11

**Authors:** Yoshiko Iwai, Hiroaki Hemmi, Olga Mizenina, Shoko Kuroda, Koji Suda, Ralph M. Steinman

**Affiliations:** 1 Laboratory of Cellular Immunology and Physiology, The Rockefeller University, New York, New York, United States of America; 2 Medical Top Track (MTT) Program, Medical Research Institute, Tokyo Medical and Dental University, Tokyo, Japan; Federal University of São Paulo, Brazil

## Abstract

Rapid proliferation is one of the important features of memory CD8^+^ T cells, ensuring rapid clearance of reinfection. Although several cytokines such as IL-15 and IL-7 regulate relatively slow homeostatic proliferation of memory T cells during the maintenance phase, it is unknown how memory T cells can proliferate more quickly than naïve T cells upon antigen stimulation. To examine antigen-specific CD8^+^ T cell proliferation in recall responses in vivo, we targeted a model antigen, ovalbumin(OVA), to DEC-205^+^ dendritic cells (DCs) with a CD40 maturation stimulus. This led to the induction of functional memory CD8^+^ T cells, which showed rapid proliferation and multiple cytokine production (IFN-γ, IL-2, TNF-α) during the secondary challenge to DC-targeted antigen. Upon antigen-presentation, IL-18, an IFN-γ-inducing factor, accumulated at the DC:T cell synapse. Surprisingly, IFN-γ receptors were required to augment IL-18 production from DCs. Mice genetically deficient for IL-18 or IFN-γ-receptor 1 also showed delayed expansion of memory CD8^+^ T cells in vivo. These results indicate that a positive regulatory loop involving IFN-γ and IL-18 signaling contributes to the accelerated memory CD8^+^ T cell proliferation during a recall response to antigen presented by DCs.

## Introduction

Memory CD8^+^ T cells are an important component of acquired immunity to viruses and other pathogens [1]–[3]. Memory T cells can persist for extended periods, and they respond more rapidly and more vigorously than naïve T cells when they reencounter the same antigen. Although several cytokines such as IL-15 and IL-7 regulate basal, relatively slow, homeostatic proliferation of memory T cells during the maintenance phase [4]–[8], it is unknown how memory T cells can proliferate more quickly than naïve T cells upon antigen re-encounter.

Memory T cells have been divided into “central” memory (T_CM_) and “effector” memory (T_EM_) subsets [2], [9]. T_CM_ express CD62L and CCR7, which allow efficient homing to lymph nodes, whereas T_EM_ lack expression of these lymph node-homing receptors and are located in the blood, spleen, and nonlymphoid tissues. T_EM_ exhibit immediate effector activity, such as the production of IFN-γ and exertion of cytotoxic activity, whereas T_CM_ have greater proliferative potential upon antigen re-encounter. Understanding the development of memory CD8^+^ T cells in response to safe forms of microbial and tumor antigens should be valuable for identifying correlates of protective immunity and the design of effective vaccines.

Immunization of mice with DCs that have been prepared and loaded with antigen in vitro generates antigen-specific CD8^+^ memory T cells [10], [11], while in vivo depletion of DCs reduces the capacity of mice to generate both primary and memory CD8^+^ T cell responses [12]. Recently, it has become feasible to selectively target antigens to DCs within intact lymphoid tissues and then examine the function of these antigen presenting cells directly in vivo, e.g., by incorporating the antigen within monoclonal antibodies (mAbs) to DEC-205/CD205, an endocytic receptor that is abundant on DCs within the T cell areas of lymphoid organs [13]–[15]. Antigen targeting results in two distinct types of primary response in the first 2–3 weeks following immunization: deletional T cell tolerance in the steady state, and T cell immunity following ligation of CD40 and the differentiation or maturation of the DCs [14], [15]. In the latter instance, the directed delivery of antigens to DCs greatly amplifies the efficiency and magnitude of the T cell response to peptides and protein antigens relative to nontargeted antigen [16]–[18], making it attractive to use directed antigen delivery to analyze the function of DCs in establishing memory in a naïve animal using defined antigens and maturation stimuli.

In this study we examined the generation of memory CD8^+^ T cells from the endogenous repertoire of normal mice following in vivo delivery of ovalbumin (OVA) via a mAb to DEC-205. We show that delivery of OVA to DEC-205^+^ DCs with CD40 ligation induces functional memory CD8^+^ T cells. Interestingly, IFN-γ signaling stimulates DCs to produce IL-18, which was originally identified as a stimulator of IFN-γ production [19], [20]. We show that this positive regulatory loop of IFN-γ-IL-18-signaling plays an important role in the acceleration of memory CD8^+^ T cell expansion during a secondary response to antigen presented by DCs.

## Results

### DEC-205 targeting with CD40 ligation expands memory CD8^+^ T cells

**Figure 1:**
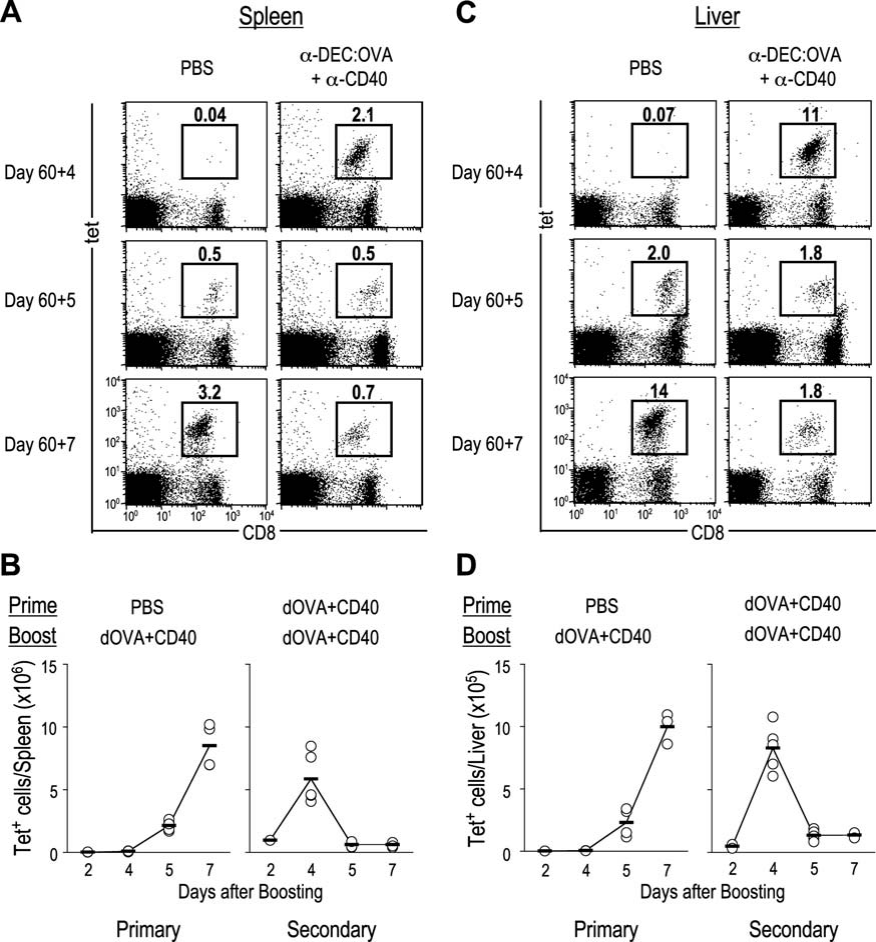
DEC-205 targeting in the presence of agonistic anti-CD40 generates memory CD8^+^ T cells. (A–D) B6 mice were primed with PBS or anti-DEC:OVA+anti-CD40, and boosted with anti-DEC:OVA+anti-CD40 at d 60. (A, C) Frequency of OVA-specific CD8^+^ T cells in spleen (A) and liver (C) at day 4, 5, and 7 after booster immunization. Numbers are % of lymphocytes. Representative of five experiments. (B, D) Total number of OVA-specific CD8^+^ T cells per spleen (B) and liver (D) after boosting, where each symbol represents data from different experiments.

To examine whether the targeted delivery of OVA to DEC-205^+^ DCs can induce memory CD8^+^ T cells, C57BL/6(B6) mice were primed with a single dose of anti-DEC-205:OVA protein in the presence of the agonistic anti-CD40 mAb, 1C10 [21], and 60 days later, the mice were boosted with anti-DEC:OVA+anti-CD40. At various time points after boosting, splenocytes were isolated and stained with MHC tetramer (Fig. 1A). In a primary response (in mice primed with PBS), OVA-specific CD8^+^ T cells appeared at day 5 and reached a peak at day 7. In recall responses (in mice primed with anti-DEC:OVA+anti-CD40), OVA-specific CD8^+^ T cells reached a peak more rapidly in spleen, at day 4. Fig. 1B summarizes the total numbers of tetramer^+^ cells per spleen in several experiments and shows the more rapid secondary response. The rapid kinetics of the antigen-specific CD8^+^ T cell response was also observed in liver (Fig. 1C and D). The secondary expansion of tetramer^+^ CD8^+^ T cells was not observed in the absence of anti-CD40 during booster immunization (data not shown). These results indicate that delivery of OVA to DEC-205^+^ DCs in the presence of CD40 ligation induces expansion of tetramer binding memory CD8^+^ T cells in both spleen and liver, and that these T cells respond rapidly to antigen rechallenge.

### DEC-205 targeting with CD40 ligation induces memory CD8^+^ T cells in multiple peripheral tissues

**Figure 2:**
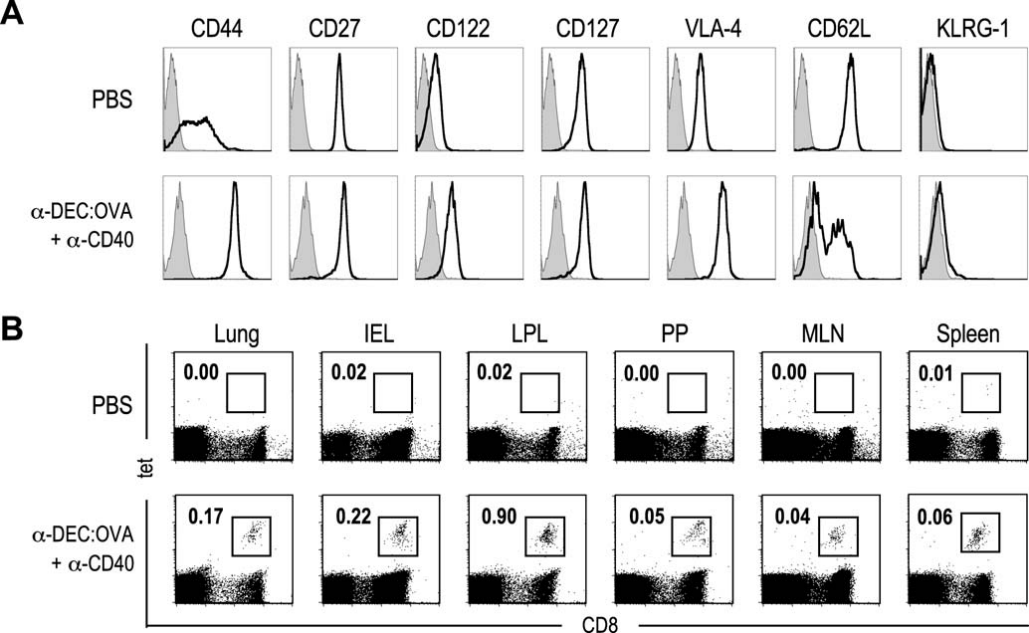
Characterization of memory CD8^+^ T cells following DEC-205 targeting of OVA. (A) Phenotype of OVA-specific memory CD8^+^ T cells at d 60 after priming with anti-DEC:OVA+ anti-CD40. OVA-specific cells were enriched using MACS sorting as described in the methods and gated as tet^+^ CD8^+^ cells. Naïve cells are not tetramer binding cells and were gated as CD44^low^ CD8^+^ cells. (B) Memory CD8^+^ T cells were identified in the indicated tissues: lung, intestine (IEL, LPL), Peyer's patches (PP), mesenteric lymph nodes (MLN), and spleen at d 60 after priming with anti-DEC:OVA+anti-CD40. Numbers are % of lymphocytes.

We phenotyped the CD8^+^ tetramer^+^ T cells 60 days after immunization with anti-DEC:OVA+ anti-CD40. The tetramer binding cells had a memory phenotype, i.e., CD27^+^, CD127^+^ with higher levels of CD44, CD122 and VLA-4 than naïve CD8^+^ T cells (Fig. 2A). They were mixture of two populations, CD62L high and low (Fig. 2A). After 60 days of priming with anti-DEC:OVA+anti-CD40 (but prior to boosting) tetramer^+^ cells were also detected in mucosal tissues such as lung and intestinal epithelium and lamina propria, and also in Peyer's patches (PP), mesenteric lymph nodes (MLN), and spleen (Fig. 2B). These memory cells were not found in peripheral tissues if mice were primed with anti-DEC:OVA without anti-CD40 (data not shown). Therefore targeting of OVA to DCs in the presence of anti-CD40 generates memory CD8^+^ T cells in multiple peripheral tissues.

### Memory CD8^+^ T cells can proliferate more rapidly than naïve T cells

**Figure 3:**
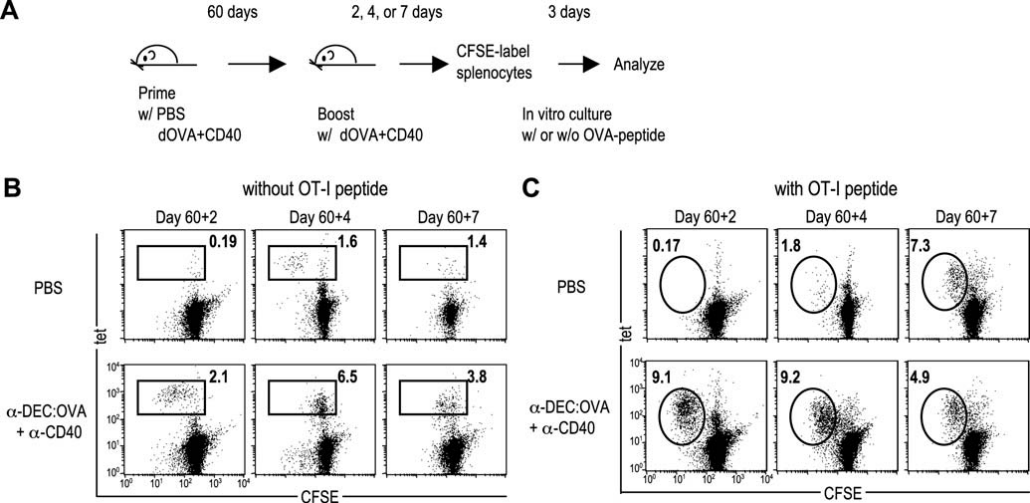
Proliferative capacity of memory CD8^+^ T cells. (A) C57BL/6 mice were primed with PBS or anti-DEC:OVA+anti-CD40, and boosted with anti-DEC:OVA+anti-CD40 at day 60. At day 2, 4, and 7 after booster immunization, splenocyes were isolated, labeled with CFSE, and then cultured without (B) or with (C) OVA(257-264) peptide. 3 days after in vitro culture, OVA-specific CD8^+^ T cell proliferation was examined by staining splenocytes with anti-CD8 and K^b^/OVA257-264 tetramer. (B) Autonomous proliferation of OVA-specific T cells in the absence of OVA peptide. Plots were gated on CD8^+^ cells. Numbers indicate the percentage of tetramer^+^ cells in CD8^+^ cells. (C) Ag-driven proliferation of OVA-specific T cell in the presence of OVA-peptide. Plots were gated on CD8^+^ cells. Numbers indicate the percentage of CFSE low tetramer^+^ cells in CD8^+^ cells.

To compare the proliferative capacity of naive and memory T cells, mice were primed with PBS or anti-DEC:OVA+anti-CD40, and boosted with anti-DEC:OVA+anti-CD40 at day 60. At day 2, 4, and 7 after boosting, splenocytes were isolated, labeled with CFSE, and then cultured in vitro for 3 days in the absence or presence of OVA257-264 peptide (Fig. 3A). T cell proliferation was monitored by CFSE dilution. It has been shown that, following CD8^+^ T cell activation, daughter cells continue to divide in the absence of further antigenic stimulation [22], [23]. Fig.3B shows T cell division in the absence of added antigen in vitro, whereas Fig.3C shows expansion when the cells re-encounter the same antigen in culture. In the mice primed with anti-DEC:OVA+anti-CD40, T cell division was observed at day 2 without further addition of antigen, but it stopped at day 4 after boosting (Fig. 3B). However, these memory T cells had greater proliferative capacity, when they reencountered OVA in culture at day 4 and 7 after boosting (Fig. 3C). In the primary response (in mice primed with PBS), T cell division was detectable at day 4 but not at day 7 (Fig. 3B), and antigen-driven expansion was observed at later time point, at day 7 (Fig. 3C). These data indicate that memory T cells can proliferate more rapidly than naïve T cells.

### Memory CD8^+^ T cells primed with anti-CD40 produce multiple cytokines

**Figure 4:**
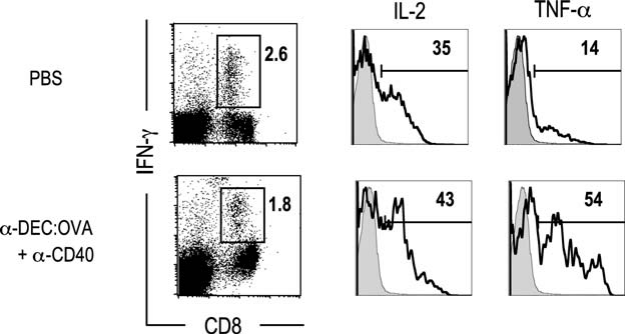
Memory CD8^+^ T cells primed with anti-CD40 produce multiple cytokines. Mice were primed with PBS or anti-DEC:OVA+anti-CD40, and boosted with anti-DEC:OVA+anti-CD40 at day 60. At the peak of the secondary response (at d 7 after boosting in PBS-primed mice; at d 4 in anti-DEC:OVA+anti-CD40-primed mice), splenocytes were stimulated with OVA(257-264) peptide and the production of IFN-γ, IL-2 and TNF-α were examined by intracellular staining. Representative of two experiments.

Next we compared the capacity of primed CD8^+^ T cells to produce cytokines during a secondary or memory response (Fig. 4). Mice were primed with PBS, or anti-DEC:OVA+anti-CD40, and boosted with anti-DEC:OVA+anti-CD40 at day 60. The OVA-specific CD8^+^ IFN-γ^+^ T cells that had been primed with anti-DEC:OVA+anti-CD40 allowed for strong IL-2 and TNF-α production upon secondary challenge. In the primary response (mice primed with PBS), the OVA-specific CD8^+^ IFN-γ^+^ T cells also produced some IL-2 and TNF-α although much less than observed with memory cells. Therefore priming with anti-DEC:OVA and agonistic anti-CD40 mAb generates memory cells with the capacity to produce high levels of multiple cytokines.

### Memory CD8^+^ T cells primed with anti-CD40 exhibit direct cytotoxicity and protective immunity

**Figure 5:**
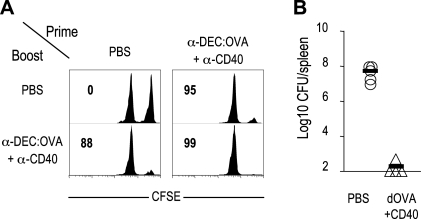
Memory CD8^+^ T cells primed with anti-CD40 show direct cytotoxicity and protective immunity. (A) In vivo CTL activity. C57BL/6 mice were primed with PBS or anti-DEC:OVA+anti-CD40, and boosted with PBS or anti-DEC:OVA+anti-CD40 at day 60. 5 d after boosting, CTL activity was determined by challenge with CFSE^hi^ OVA257-264 peptide-loaded splenocytes. Numbers indicate % of specific killing. (B) Bacterial numbers in spleen at day 4 after infection. B6 mice were primed with PBS or anti-DEC:OVA+anti-CD40, and 60 d later, injected i.v. with 1×10^5^ virulent LM-OVA (four mice per group).

To examine the in vivo cytolytic function of the memory T cells, mice were primed with PBS or anti-DEC:OVA+anti-CD40, and boosted with PBS or anti-DEC:OVA+anti-CD40 at d 60 (Fig. 5A). Before boosting and at day 5 after boosting, the mice were injected with a mixture of syngeneic splenocytes pulsed with or without OVA257-264 peptide, and the elimination of peptide-pulsed splenocytes (CFSE^hi^) was examined by flow cytometry. In the absence of boosting (Fig. 5A, upper row), strong cytotoxic activity was only observed following priming with anti-DEC:OVA+anti-CD40, while after boosting, both primary and secondary responses were associated with cytotoxic activity (Fig. 5A, lower row).

To determine if the improved effector functions of memory CD8^+^ T cells was associated with protection, mice were immunized with PBS or anti-DEC:OVA+anti-CD40, and 60 days later, the mice were challenged with 1×10^5^ colony-forming units (CFU) of virulent *Listeria monocytogens* expressing OVA (LM-OVA) (Fig. 5B). Mice immunized with anti-DEC:OVA+anti-CD40 were protected, but mice immunized with PBS were not. Therefore memory T cells generated by priming with anti-CD40 exert stronger effector functions and protective immunity.

### IL-18 accumulates at the DC:T cell synapse and regulates antigen-specific CD8^+^ T cell proliferation in both primary and memory responses

**Figure 6:**
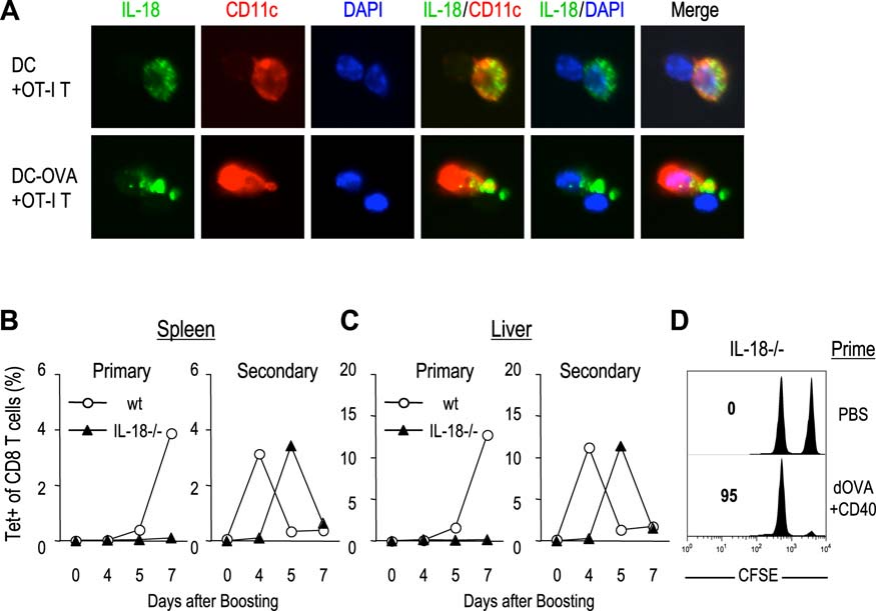
IL-18 accumulates at the DC:T synapse and regulates CD8^+^ T cell proliferation. (A) OVA(257-264) peptide-pulsed or unpulsed DCs were cultured with OT-I transgenic CD8^+^ T cells, and stained with anti-IL-18 (green) and anti-CD11c (red). Nuclei were counterstained with DAPI (blue). (B, C) IL-18^−/−^ and wild type mice were primed with PBS or anti-DEC:OVA+anti-CD40, and boosted with anti-DEC:OVA+anti-CD40 at d 60. At day 0, 4, 5, and 7 after boosting, lymphocytes were isolated from spleen (B) and liver (C), and stained with Kb/OVA257-264 tetramer. (B and C) Percentage of tetramer^+^ cells in CD8^+^ T cells. (D) In vivo CTL activity. IL-18^−/−^ mice were primed with PBS, or anti-DEC:OVA+anti-CD40, without booster immunization. At d 60, CTL activity was determined by challenge with CFSE^hi^ OVA257-264 peptide-loaded splenocytes. Numbers indicate the % of specific killing. Representative of two experiments.

IL-18 is a proinflammatory cytokine, which was originally identified as an IFN-γ-inducing factor [19], [20]. It has been reported that memory T cells express high levels IL-18 receptor (IL-18R) on memory T cells, and that IL-18 can induce bystander proliferation of memory phenotype CD8^+^ T cells [24]–[26]. Like IL-1β, IL-18 lacks a secretory signal sequence [27]–[29]. In the steady state, IL-18 was expressed in DCs at the protein (Fig. 6A) and mRNA levels (data not shown). During antigen presentation to T cells, IL-18 localized to the immunologic synapse (Fig. 6A). When the OT-I transgenic CD8^+^ T cells were cultured with DCs without peptide, IL-18 was distributed in the cytosol (Fig. 6A, upper row), but when OT-I T cells were cultured with OVA(257-264) peptide-pulsed DCs for 30 min, the IL-18 became polarized in the DCs and faced the contact region or synapse with T cells (Fig. 6A, lower row).

To examine the role of IL-18 in antigen-specific primary and memory responses, IL-18^−/−^ and wild type mice were primed with PBS or anti-DEC:OVA+anti-CD40 and boosted at day 60 (Fig. 6B and C). In the primary response, significant antigen-specific CD8^+^ T cell proliferation was observed in neither spleen (Fig. 6B) nor liver (Fig. 6C) of IL-18^−/−^ mice. Surprisingly, Ag-specific T cell proliferation in the secondary response was observed in IL-18^−/−^ mice, but the expansion was delayed compared with wild type mice. Despite the impaired expansion of memory T cells in IL-18^−/−^ mice, the cells showed direct cytotoxic activity (Fig. 6D). These results suggest that effector memory develops in IL-18^−/−^ mice, but T cell proliferation both in primary and memory responses was impaired in the absence of IL-18.

### IFN-γ stimulates IL-18 production from DCs and enhances the expansion of memory CD8^+^ T cells

**Figure 7:**
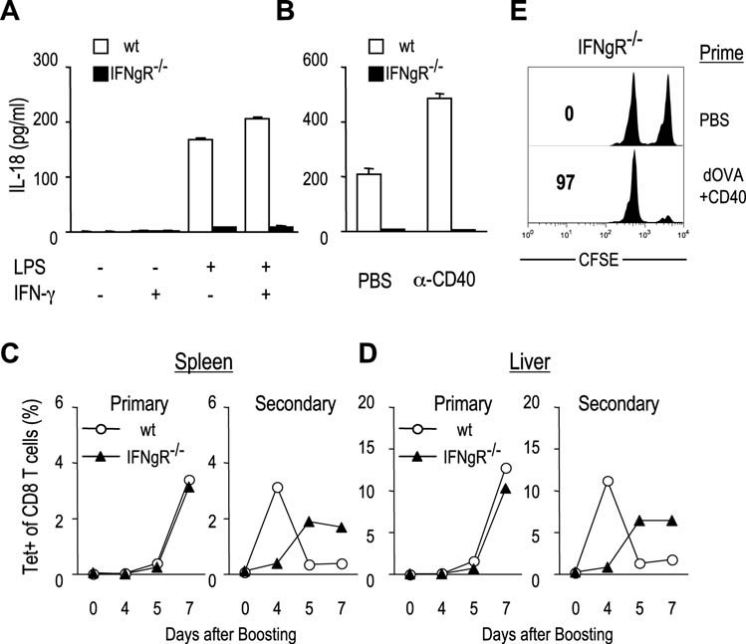
IFN-γ signaling regulates the expansion of memory CD8^+^ T cells. (A, B) IL-18 production from DCs. (A) DCs isolated from spleen of IFN-γR1^−/−^ and wild type mice were stimulated with LPS in the presence or absence of IFN-γ for 24 hr. (B) IFN-γR1^−/−^ and wild type mice were injected with PBS or anti-CD40 and 1hr later, DCs were isolated and cultured in the presence of LPS for 24 hr. The culture supernatants were harvested and IL-18 production was measured by ELISA. (C, D) IFN-γR1^−/−^ and wild type mice were primed with PBS or anti-DEC:OVA+anti-CD40, and boosted with anti-DEC:OVA+anti-CD40 at day 60. At day 0, 4, 5, and 7 after boosting, lymphocytes were isolated from spleen (C) and liver (D), and stained with Kb/OVA257-264 tetramer. (C and D)Percentage of tetramer^+^ cells in CD8^+^ T cells. (E) In vivo CTL activity. IFN-γRI^−/−^ mice were primed with PBS or anti-DEC:OVA+anti-CD40, without booster immunization. At d 60, CTL activity was determined by challenge with CFSE^hi^ OVA257-264 peptide-loaded splenocytes. Numbers indicate the % of specific killing. Representative of two experiments.

It has been reported that IL-18 can stimulate IFN-γ production from lymphocytes including T cells, B cells, and NK cell [20], [30], [31]. Interestingly, we found that IL-18 production from DCs decreased in the absence of IFN-γ signaling. Upon stimulation with LPS, DCs from IFN-γ receptor 1(IFN-γR1)^−/−^ mice showed much lower IL-18 production than those in wt mice (Fig. 7A). In vivo administration of anti-CD40 mAb enhanced IL-18 production from DCs in wt mice but not in IFN-γR1^−/−^ mice (Fig. 7B). These results suggest the positive loop of IL-18-IFN-γ signaling, in which IFN-γ stimulates IL-18 production from DCs.

Recently, Badovinac et al. have shown that priming without early inflammation, especially in the absence of IFN-γ, accelerates the development of memory CD8^+^ T cells [11]. To examine the role of IFN-γ in CD8^+^ T cell memory responses, IFN-γRI^−/−^ and wild type mice were primed with PBS or anti-DEC:OVA+anti-CD40, and boosted with anti-DEC:OVA+ anti-CD40 at day 60 (Fig. 7C, and D). At day 0, 4, and 5 after boosting, splenocytes were harvested and stained with Kb/OVA257-264 tetramer (Fig. 7C). Unlike IL-18^−/−^ mice, the expansion of tetramer^+^ CD8^+^ T cells in primary response was normal in IFN-γR1^−/−^ mice. However, in the secondary response (mice primed with anti-DEC:OVA+anti-CD40), the expansion of tetramer^+^ CD8^+^ T cells was delayed in IFN-γRI^−/−^ mice compared with wild type mice. Similar kinetics of tetramer^+^ CD8^+^ cells was observed in the liver (Fig. 7D). Similar to IL-18^−/−^ mice, memory IFN-γRI^−/−^ T cells showed direct cytotoxic activity, which was identical to that of wild type memory T cells (Fig. 7E and Fig. 5A). Taken together, our results suggest that IFN-γ signaling induces IL-18 production from DCs and accelerates the proliferation of memory CD8^+^ T cells rather than naïve T cells.

## Discussion

The DEC-205^+^ or CD8^+^ subset of splenic DCs is known to be specialized for the presentation of antigens on MHC class I products [32]–[34]. We now find that this receptor and subset, when selectively targeted in vivo with OVA within anti-DEC-205 monoclonal antibody, is able to generate functional CD8^+^ memory T cells depending upon the coadministration of a CD40 signal at the time of antigen presentation.

Delivery of the OVA antigen to DEC-205^+^ DC subset in the presence of agonistic CD40 mAb induces effector-type memory CD8^+^ T cells that respond to antigen re-encounter rapidly but for a short-term. These effector cells populate peripheral tissues including mucosal tissues (lung, intestinal epithelium) and mucosal associated lymphoid organs (Peyer's patch and mesenteric lymph node) both before and after the secondary response. The memory T cells are functional, producing IL-2, IFN-γ, and TNF-α and exerting direct cytotoxic activity. In addition, DEC-205 targeting with simultaneous CD40 ligation provides protective immunity. These data suggest the value of CD40 ligation for DEC-205^+^ DCs to generate protective memory CD8^+^ T cells.

We showed that IL-18 accumulates at the DC:T synapse during antigen-presentation. Although it has been reported that IL-18 can induce bystander proliferation of memory CD8^+^ T cells [25], [26], based on the manner of IL-18 secretion, we have reasoned that IL-18 might regulate antigen-specific T cell proliferation. As expected, IL-18 regulates antigen-specific CD8^+^ T cell proliferation both in primary and memory responses. In the absence of IL-18 signaling, obvious CD8^+^ T cell proliferation was not observed in primary responses, but memory CD8^+^ T cells with direct cytotoxic activity were generated. However, these IL-18^−/−^ memory cells showed delayed expansion compared with wild type memory T cells. These results suggest that IL-18 is not critical for generation of memory CD8^+^ T cells, but enhances antigen-specific CD8^+^ T cell proliferation both in primary and memory responses.

**Figure 8:**
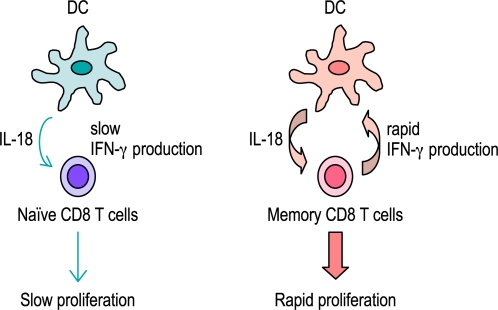
Model of a positive regulatory loop of IFN-γ-IL-18 signaling in memory CD8^+^ T cell expansion. Unlike naïve CD8^+^ T cells, memory CD8^+^ T cells can provide an early source of IFN-γ to stimulate more IL-18 production from DCs. This positive loop of IFN-γ-IL-18 signaling may play an amplifying role in the acceleration of CD8^+^ T cell expansion in memory response rather than in primary response.

Although IL-18 was originally identified as a stimulator of IFN-γ production [19], [20], our results show an positive loop of IL-18-IFN-γ signaling, in which IFN-γ can also stimulate IL-18 production from DCs. Interestingly, IFN-γR1^−/−^ mice and IL-18^−/−^ mice show different patterns of T cell proliferation. In the absence of IFN-γ signaling, the expansion of antigen-specific CD8^+^ T cells was normal in primary response but delayed in secondary response. These results indicate that IFN-γ signaling plays an amplifying role in the acceleration of memory CD8^+^ T cell proliferation. Unlike naïve CD8^+^ T cells, memory CD8^+^ T cells can provide an early source of IFN-γ to stimulate more IL-18 production from DCs. Probably, this positive loop of IFN-γ-IL-18 signaling may function in recall response rather than in primary response (Fig. 8).

Our observation that IFN-γ accelerates the expansion of memory CD8^+^ T cells seems to conflict with the report by Badovinac et al. that IFN-γ prevents the generation of memory CD8 T cells [11]. However, there are some differences in the experimental system. Badovinac et al studied in vitro cultured mature DCs, while we addressed DC function in vivo. In addition, they analyzed the memory response at a later time point (at day 6 after boosting), while we examined the kinetics of memory T cells including earlier time points. We observed more antigen-specific CD8^+^ T cells in wild type mice than IFN-R1γ^−/−^ mice at day 4, but the results were the opposite at day 5 and 7 because of the delayed T cell expansion in IFN-R1γ^−/−^ mice.

Unlike IL-15 and IL-7, which regulate basal, relatively slow, homeostatic proliferation of memory T cells during the maintenance phase [4]–[8], the IFN-γ-IL-18 signaling pathway controls the rapid proliferation of memory CD8^+^ T cells during the second effector phase in concert with antigen-presentation by dendritic cells. Our findings will help in research to develop vaccines to induce antigen-specific T cells with a capacity to respond quickly against viruses and other pathogens.

## Materials and Methods

### Mice

C57BL/6 (B6) and BALB/c mice were from Taconic Farms (Germantown, NY). IL-18 -deficient (−/−) mice and IFN-γR1^−/−^ mice (B6 background) were from The Jackson Laboratory. Females at 8–10 wk of age were used and maintained under specific pathogen-free conditions in accordance with Institutional Care and Use Committee guidelines of The Rockefeller University.

### Antibodies and peptides

We purchased from BD Biosciences: anti-CD27-FITC, anti-CD8-APC, anti-CD8-FITC, anti-CD44-PE, anti-CD49d-FITC, anti-CD62L-FITC, anti-CD122-FITC, anti-IFN-γ-PE, anti-IFN-γ-APC, anti-IL-2-PE, and anti-TNF-α-PE; from e-Bioscience: anti-CD127-FITC and anti-CD127-APC; from Southern Biotechnology: anti-KLRG-1-FITC, from Cedarlane Labs: anti-DEC205-PE; and from Santa Cruz: goat polyclonal anti-IL-18. OVA257-264 (SIINFEKL) peptide was synthesized by the Proteomics Resource Center (The Rockefeller University).

### Generation of Hybrid Antibodies

Anti-DEC:OVA was generated as described [18]. Hybrid antibodies were transiently expressed in 293T cells using calcium phosphate. Antibodies were purified on Protein G columns (GE Healthcare) and subject to detoxification with Detoxi-Gel™ Endotoxin Removing Gel (PIERCE). Removal of endotoxin was verified with Limulus Amebocyte Lysate (LAL) QCL-1000 (CAMBREX).

### Immunization

Mice were primed i.p. with 5 µg of anti-DEC:OVA in the presence of 30 µg of anti-CD40 mAb (clone 1C10).

### Bacterial Infections

*L. monocytogenes* expressing the OVA gene (LM-OVA) was provided by Dr. Eric G. Pamer (Sloan-Kettering Institute, New York, NY) and Dr. Hao Shen (University of Pennsylvania School of Medicine, Philadelphia, PA). The bacteria were grown in brain heart infusion (BHI) broth and injected i.v. Colony-forming units were assessed by plating 10-fold dilutions on BHI agar containing 1 µg/ml erythromycin.

### Isolation of lymphocytes from nonlymphoid tissues

Intrahepatic lymphocytes were isolated from livers as described [35]. Small intestinal intraepithelial lymphocytes (IEL) and lamina propria lymphocytes (LPL) were isolated as described [36], [37]. For preparation of lung lymphocytes, the lungs were perfused with 10 ml of PBS, removed, minced and incubated in 1 ml of RPMI1640 with 5% fetal calf serum (FCS) containing 125 U/ml of collagenase D (Roche) and 60 U/ml of Dnase I (Roche) for 25 min at 37°C. Mononuclear cells were purified by Percoll gradient centrifugation.

### Isolation of CD11^+^ DCs and TCR transgenic CD8^+^ T cells

DCs were purified from collagenase-trated spleens by positive selection with anti-CD11c microbeads (Miltenyi Biotech). T cells were purified from spleens and lymph nodes of TCR Tg mice using T cell enrichment columns (R&D). T cells were incubated with anti-CD4 microbeads and CD8 T cells purified by negative selection with MACS separation columns (Miltenyi Biotech).

### MHC tetramer staining

Lymphocytes (1×10^6^) were blocked Fc block (clone 2.4G2, BD Biosciences) and unlabeled streptavidin (Molecular Probes). Cells were washed and then stained with K^b^/OVA257-264 tetramer (kindly provided by Dr. E. Pamer) and anti-CD8 in combination with anti-CD27, anti-CD44, anti-CD49d, anti-CD62L, anti-CD122, anti-CD127, or anti-KLRG-1, for 1 hr at 4°C. 200,000–400,000 events were acquired on a FACSCalibur™ (Becton Dickinson). Data were analyzed using CellQuest™ (Becton Dickinson) and FlowJo (TreeStar) software.

### Enrichment of tetramer^+^ cells

T cells were purified from collagenase-treated spleens by negative selection using T cell enrichment columns (R&D). T cells were incubated with PE-labeled K^b^/OVA257-264 tetramer, followed by anti-PE microbeads (Miltenyi Biotech).

### Intracellular cytokine staining

Lymphocytes (5×10^6^) were incubated with 2 µM of OVA (257-264) peptide in the presence of 2 µg/ml of anti-CD28 mAb (clone 37.51) for 6 hr. Brefeldin A (10 µg/ml; Sigma-Aldrich) was added for the last 4 hr of culture. After surface staining with anti-CD8 and anti-CD62L, cells were stained with anti-IFN-γ using Cytofix/Cytoperm kit (BD Biosciences) according to manufacturer instructions. In some experiments, cells were stained with anti-CD8 and anti-IFN-γ in combination with anti-IL-2 or TNF-α. 2–4×10^5^ events were acquired on a FACSCalibur™ (Becton Dickinson) as described above.

### Ex vivo proliferation

Splenocytes at 1×10^7^ cells/ml were labeled with 0.05 µM CFSE for 10 min at 37°C. Cells were washed and cultured with or without 2 µM of OVA (257-264) peptide. After 60 hr, cells were stained with anti-CD8 and K^b^/OVA257-264 tetramer and evaluated by flow cytometry for CFSE dilution.

### In vivo CTL assay

C57BL/6 splenocytes were incubated for 1 hr at 37°C with or without 10 µM of OVA (257-264) peptide and labeled for 10 min at 37°C with carboxyfluorescein diacetate succinimidyl diester (CFSE, Molecular Probes) at 5 µM (CFSE^hi^, peptide-labeled splenocytes) or 0.5 µM (CFSE^lo^, splenocytes without peptide). Immunized or naïve control mice were injected i.v. with 10^7^ cells of each fraction. Splenocytes were isolated 18 hr later, and analyzed by flow cytometry. Specific cytotoxicity was calculated with the following equation: cytotoxicity (%) = [1−(ratio immune/ratio naive)]×100. Ratio = percentage of CFSE^hi^/percentage of CFSE^lo^.

### Conjugate formation and immunofluorescene

Conjugates between T cells and DCs were formed by mixing OT-I transgenic CD8 T cells and OVA (257-264) peptide-pulsed (1 µg/ml) or unpulsed DCs at a 2∶1 ratio and a quick centrifugation at 300×*g* for 30 sec to initiate cell–cell contact. Cells were incubated at 37°C, 5% CO2 for 30 min. Cells were fixed and permeabilized with Cytofix/Cytoperm (BD Biosciences) for 20 min at room temperature, and then stained with goat polyclonal anti-IL-18 in combination with hamster anti-CD11c-biotin, followed by anti-goat IgG-FITC with streptavidin-Cy3. Cells were treated with DAPI to stain nuclei and analyzed in an Olympus IX70 microscope.

### ELISA

Mice were injected i.p. with or without 30 µg of anti-CD40 mAb (clone 1C10) and1 hr later, DCs were isolated from spleen. The DCs (5×10^5^/well) were stimulated with 500 ng/ml LPS for 24 hr. The culture supernatants were harvested and IL-18 concentration was determined using a commercial ELISA lit (MBL).
